# Computer-Assisted Teaching of Skin Flap Surgery: Validation of a Mobile Platform Software for Medical Students

**DOI:** 10.1371/journal.pone.0065833

**Published:** 2013-07-23

**Authors:** David P. de Sena, Daniela D. Fabricio, Maria Helena I. Lopes, Vinicius D. da Silva

**Affiliations:** 1 Postgraduate Program in Health Sciences, Pontifícia Universidade Católica do Rio Grande do Sul (PUCRS), Porto Alegre, Brazil; 2 Department of Otorhinolaryngology, Hospital Sao Lucas, PUCRS, Porto Alegre, Brazil; 3 Department of Internal Medicine, Faculdade de Medicina, PUCRS, Porto Alegre, Brazil; 4 Department of Pathology and Radiation, Faculdade de Medicina, PUCRS, Porto Alegre, Brazil; UC Davis School of Medicine, United States of America

## Abstract

The purpose of this study was to develop and validate a multimedia software application for mobile platforms to assist in the teaching and learning process of design and construction of a skin flap. Traditional training in surgery is based on learning by doing. Initially, the use of cadavers and animal models appeared to be a valid alternative for training. However, many conflicts with these training models prompted progression to synthetic and virtual reality models. Fifty volunteer fifth- and sixth-year medical students completed a pretest and were randomly allocated into two groups of 25 students each. The control group was exposed for 5 minutes to a standard text-based print article, while the test group used multimedia software describing how to fashion a rhomboid flap. Each group then performed a cutaneous flap on a training bench model while being evaluated by three blinded BSPS (Brazilian Society of Plastic Surgery) board-certified surgeons using the OSATS (Objective Structured Assessment of Technical Skill) protocol and answered a post-test. The text-based group was then tested again using the software. The computer-assisted learning (CAL) group had superior performance as confirmed by checklist scores (p<0.002), overall global assessment (p = 0.017) and post-test results (p<0.001). All participants ranked the multimedia method as the best study tool. CAL learners exhibited better subjective and objective performance when fashioning rhomboid flaps as compared to those taught with standard print material. These findings indicate that students preferred to learn using the multimedia method.

## Introduction

The traditional form of training in surgery is to operate under the supervision of a qualified physician, in a model based on learning by doing [1]. The changing landscape of health and medical curricula, restricted contact of students with real situations and reduced availability of teachers to supervise students along with the emergence of new mobile computing platforms has encouraged a search for new alternatives for training and education [2].

Initially, the use of cadavers and animal models appeared to be a valid alternative for training. However, ethical conflicts with these training models prompted progression to synthetic and virtual reality models [3], [4]. Doubts remained as to the ability of these models to provide information and skills suitable for use in real situations [5], but they have since been objectively validated [6]–[9].

Surgical skills laboratories were conceived as an environment created using training models [7] to provide appropriate learning situations to prepare students for real intervention, thus complementing surgical training.

The improvement of technical skills in students trained in surgical labs can be validated by evaluation tools such as OSATS (Objective Structured Assessment of Technical Skills) [3]. Regarding the optimal model for training, the use of artificial models such as bench models, is often equivalent to animal models for the acquisition of surgical skills [10], [11]. The use of virtual reality and CAL (Computer Assisted Learning) facilitates the learning process and enables effective acquisition of surgical skills [12]. Students can thus learn on their own (self-learning system), regardless of the presence of an instructor to ensure good performance [13], especially when basic surgical procedures are being practiced. However, it is well established that those who receive feedback from their instructors during the learning process still have better results [14].

The surgical skill acquisition process is 75% decision making and only 25% surgical dexterity [15]. Assimilation of the geometry and design of skin flaps using a multimedia CAL (computer-assisted learning) tool can enhance student surgical skills, which can then be evaluated and validated by an OSATS [3] protocol.

The classic Limberg rhomboid flap [16], [17] was chosen to test the evaluation of skin flaps as it is a commonly used flap in the practice of plastic, dermatologic and general surgeons alike. It is also a procedure with an easy and fast paced learning curve, a crucial point to this study design. Once the efficiency of CAL methods combined with training stations is confirmed, students will have an important tool for study and training.

## Objectives

To develop, validate and evaluate the applicability of a multimedia software application ready for mobile platforms that assists in the teaching and learning process of skin flap surgery.

## Materials and Methods

### Ethics Statment

This was a prospective, randomized, controlled study approved by the Ethics Committee of the Pontifical Catholic University of Rio Grande do Sul (PUCRS), Brazil.

### Methodology

Fifth- and sixth-year medical students were invited to take part in the study. The exclusion criteria were prior experience in designing or fashioning a rhomboid flap or experience assisting, aiding, or otherwise performing the procedure.

Each student completed an informed consent form and a pretest consisting of five multiple-choice items about the subject matter. Randomization was performed by use of sealed brown envelopes delivered to students at random, without prior identification.

Two groups of 25 students were formed. Participants in each group received either a standard, text-based print article (printed text group) or laptop computers with a multimedia software application for self-education about detailed rhomboid flap making (CAL group).

The article used by the printed text group was based on a book chapter [14], chosen because it uses illustrative figures and demonstrates a simple and objective how-to method, with emphasis on key points. The article chapter was modified to describe only the classic rhomboid flap, excluding any description of other types of procedures.

The CAL module was built so as to cover the same content of the printed article. Its content was compared to the printed text, reviewed, evaluated and approved by three board-certified plastic surgeons.

Both groups were given the use of a quiet, isolated room where they could assess their respective teaching methods. No questions or dialogues between them were allowed. It took a mean time of one minute and one minute and 25 seconds for the students to read the printed material and to complete the CAL module respectively. As time available for training activities is a key variable, any tool or method capable of imparting knowledge efficiently in a short time is useful for teaching and learning efficiently. This prompted us to give students 5 minutes of study exposure prior to hands-on testing at the training stations, thus allowing the students to review the printed or the CAL material at least three times so as to provide evidence of understanding and retention of acquired knowledge for immediate use.

**Figure 1:**
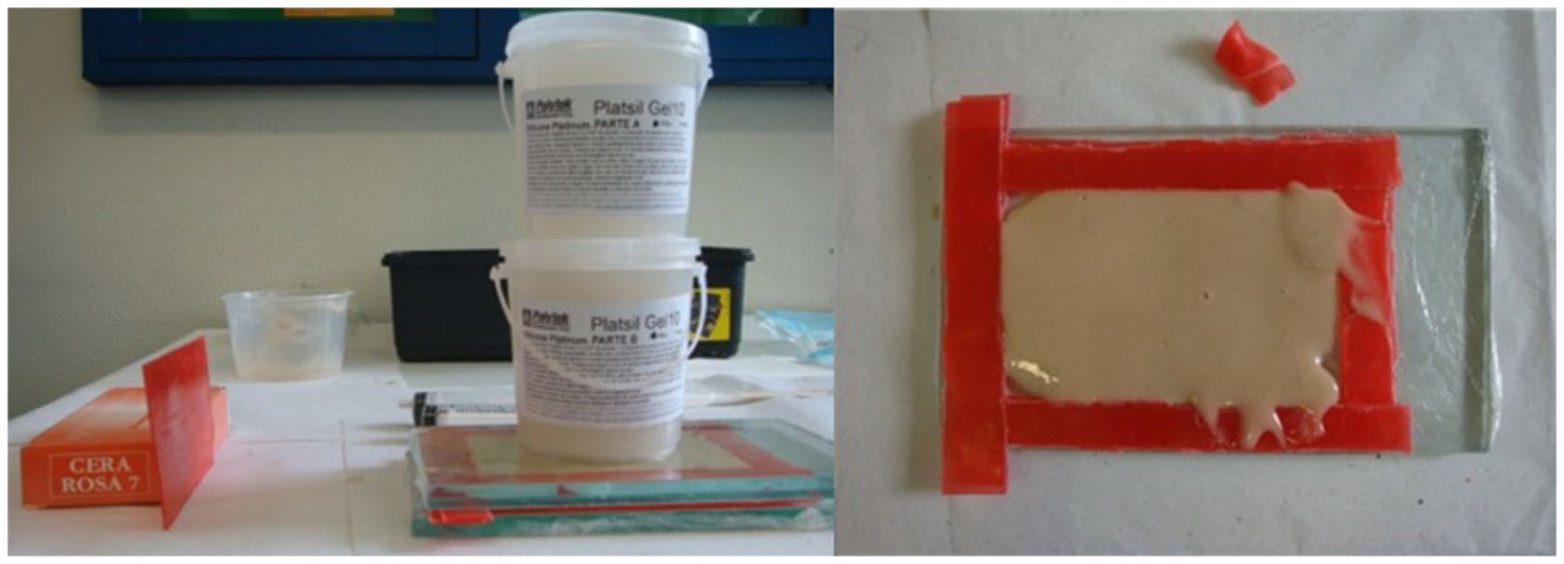
Manufacturing non-animal silicon skin.

**Figure 2:**
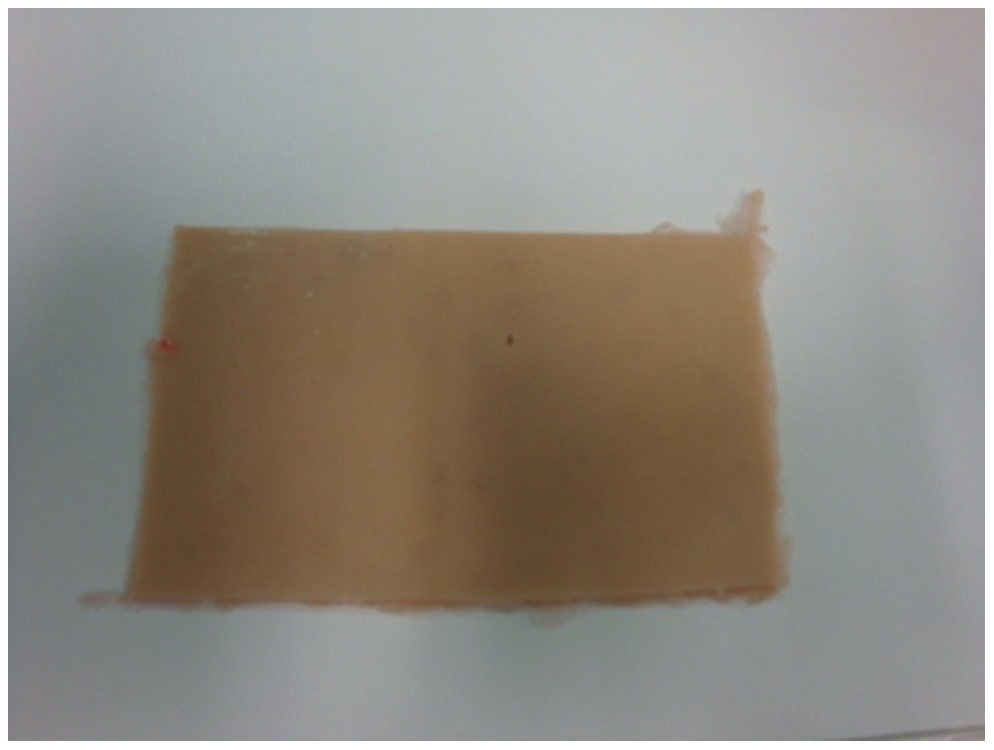
Non-animal silicon skin fragment, 4×6 cm.

**Figure 3:**
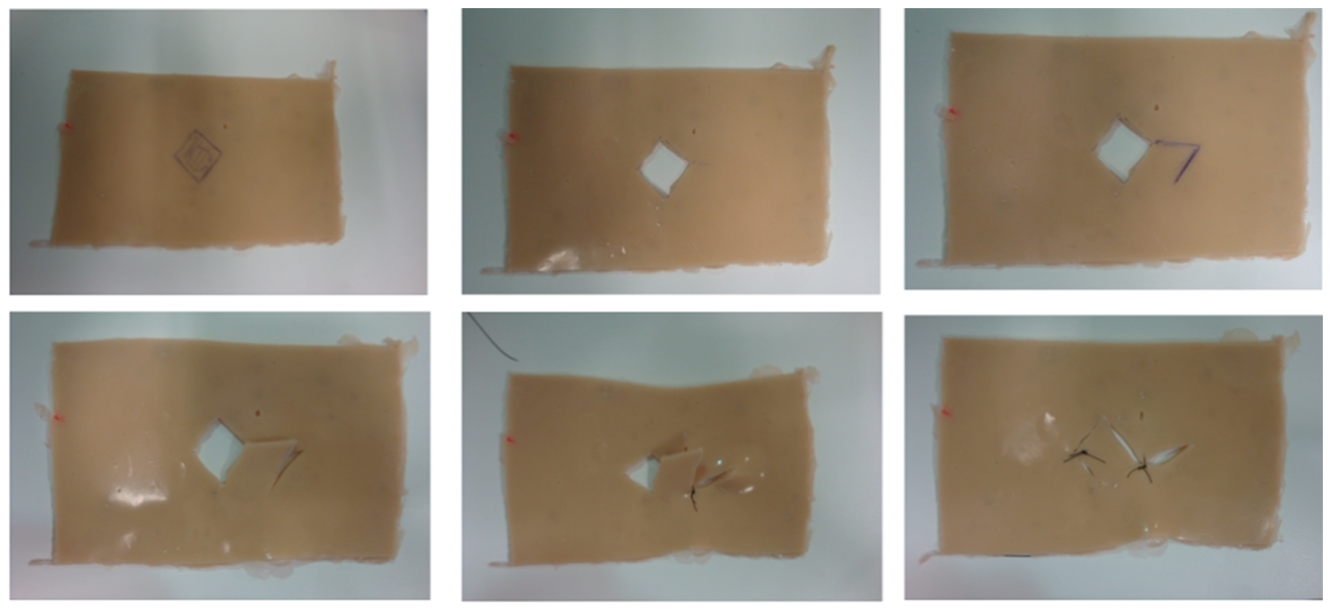
Sequence of the rhomboid skin flap.

After this period, the students were assigned to a training station to resect a simulated lesion and fashion a rhomboid flap on a skin model, as shown in Figures 1, 2 and 3, also for five minutes. The training stations contained an experimental silicon skin model, 4×6 cm in size, labeled with a circular square-centimeter central design, as shown in Figure 3, fixed to a cork board by metal staples. Students had basic surgical instruments at their disposal, 3.0 mononylon suture material, a ruler and a pen.

The performance of each student while resecting the lesion and fashioning the flap was assessed by a blinded, board-certified plastic surgeon using the OSATS [3] protocol, which consists of a checklist and a global performance assessment. The checklist was composed of 10 right-or-wrong items, where each participant received one point for each item correctly performed. The Global Assessment Scale consisted of nine descriptive items, including respect for tissue, time and motion, instrument handling, surgery flow, procedure knowledge, dexterity, visual spatial ability, overall performance and final surgical product quality, the score of which could range from one to five points. At the end of the 5-minute period, students completed a post-test consisting of the same five-item multiple-choice pretest, including what is the rhomboid flap design, the correct angles of the design, right closure, resection and which suture should be done first.

Next, the printed text group was also exposed to the multimedia software for 5 minutes and reattempted to perform the rhomboid flap at the training stations. Group participants were reassessed and completed the post-test again. The CAL group also had access to the printed text for comparison purposes, without, however, being reevaluated at the training station. At the end of the study, to determine the responsiveness to each method, all students answered a questionnaire about their post-test impressions of each method.

**Figure 4:**
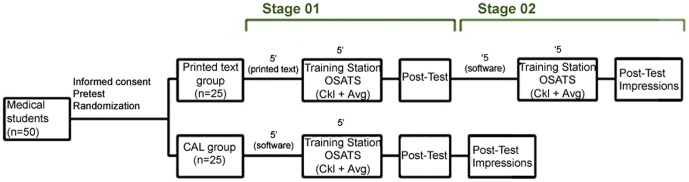
Study flowchart. n: number of students; 5′ - five minutes; OSATS - Objective Structured Assessment of Technical Skill; Ckl - checklist; Avg - Global Assessment Scale.

Figure 4 shows a schematic of the distribution of groups, procedures and timing of each of the study stages.

At the end of the process, we compared the percentage of correct answers and the scores of each group to determine which training produced the best practical results, as an expression of the acquisition of surgical skills. At the conclusion of the study, students were asked to complete a questionnaire about the quality of the teaching methods used and comment on which method they would choose as the best approach for teaching and learning.

Categorical data were described as absolute and relative frequencies, and quantitative data (scores), as mean and standard deviation. In independent groups, we used the chi-square test with Yates' continuity correction to compare proportions and the Mann–Whitney *U* test to compare scores. In matched groups, the McNemar chi-square test was used to compare proportions, and the Wilcoxon *t*-test, to compare scores. Data were analyzed using the SPSS 7.0 software (SPSS Inc, Chicago, IL, USA).

The multimedia software program was developed using Macromedia Flash MX 2004 (Adobe Systems Inc., San Jose, CA, USA) [18], based on vector animations, text and audio, with a total duration of 1 minute and 25 seconds. When the program is run, the audio narration begins, pausing automatically when the program is interrupted. Students were able to navigate freely, returning or fast-forwarding content as desired.

## Results

A total of 50 students, 29 men (58%) and 21 women (42%), took part in the study. Of these, 25 were allocated to the printed text group and 25 to the CAL group. All students completed a multiple choice pre-test which confirmed total ignorance of the rhomboid flap technique. Performance at the training stations is reported below, with comparisons between the printed-text and CAL groups and comparison of the performance of the printed text group before and after exposure to the CAL software.

**Figure 5:**
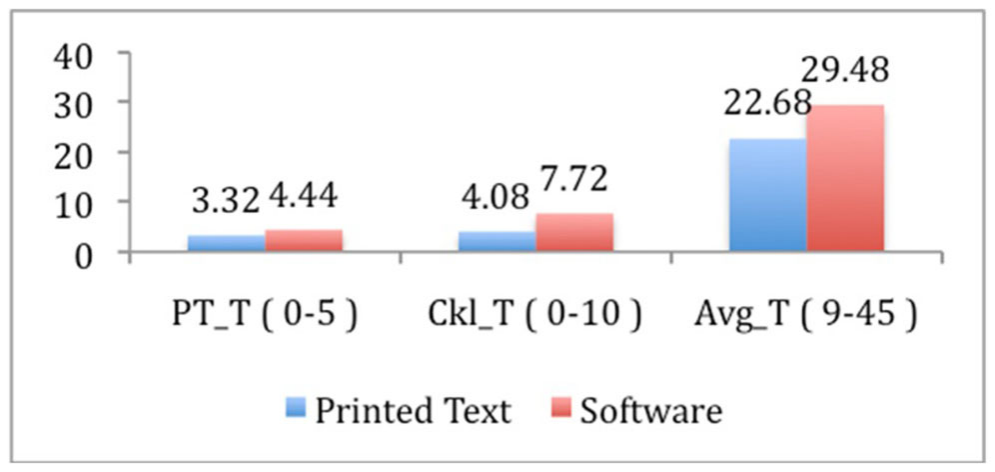
Average post-test, Checklist and Global Assessment final score. p: statistical significance calculated by the Mann-Whitney U; PT_T Mean = Mean post-test sum of items (p<0.001); Ckl_T = Mean sum of checklist items (p<0.002); Avg_T = Mean sum of overall assessment items (p<0.017).

**Figure 6:**
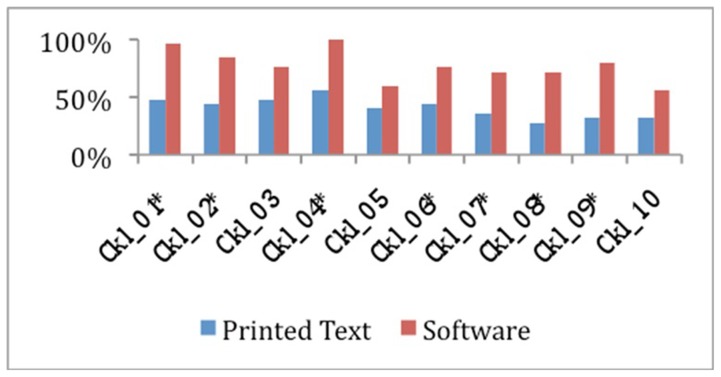
Correct response percentages for each checklist item. CKL = Check List. * p<0.05 (chi-square test with Yates' correction).

The mean raw score of all 10 checklist items was 4.08±4.0 for the printed text group vs. 7.72±2.05 for the CAL group (p<0.002), as shown in figure 5 and table 1. The average proportion of correct responses was superior in the CAL group for all items. The items that showed the greatest differences were CK_01 (flap orientation), with 48% vs. 96% (p = 0.001), CK_07 (flap position), with 36% vs. 72% (p = 0.023), and CK_09 (major sutures held first), with 32% vs. 80% (p = 0.002), as shown in Figure 6 and Table 1.

**Table 1:** Printed text group vs. CAL group.

Checklist			
Feature	Text (n = 25)	CAL (n = 25)	(p)[1]
Number of correct answers, n (%)			
Ckl_01	12 (48)	24 (96)	0.001
Ckl_02	11 (44)	21 (84)	0.008
Ckl_03	12 (48)	19 (76)	0.08
Ckl_04	14 (56)	25 (100)	0.001
Ckl_05	10 (40)	15 (60)	0.258
Ckl_06	11 (44)	19 (76)	0.043
Ckl_07	09 (36)	18 (72)	0.023
Ckl_08	07 (28)	18 (72)	0.005
Ckl_09	08 (32)	20 (80)	0.002
Ckl_10	08 (32)	14 (56)	0.154
Gross Score (0 to 10)		(p)[2]	
Ckl_T	4.08±4.0	7.72±2.05	<0.002

Global Assessment			
Score (1 to 5)		(p)[2]	
Avg_01	3.12±0.83	3.56±1.26	0.115
Avg_02	2.48±1.16	3.12±0.73	0.018
Avg_03	2.96±1.14	3.48±1.36	0.133
Avg_04	2.56±1.36	3.52±1.33	0.013
Avg_05	2.36±1.58	3.40±1.41	0.013
Avg_06	2.36±1.35	2.92±1.04	0.084
Avg_07	2.6±1.48	3.36±1.29	0.054
Avg_08	2.12±1.27	3.04±1.43	0.026
Avg_09	2.12±1.33	3.08±1.32	0.016
Score (9 to 45)			
Avg_T	22.68±10.53	29.48±9.40	0.017

Post-Test			
Feature	Text (n = 25)	CAL (n = 25)	(p)[1]
Number of correct answers, n (%)			
PT_01	21 (84)	25 (100)	0.118
PT_02	22 (88)	25 (100)	0.234
PT_03	11 (44)	23 (92)	0.001
PT_04	10 (40)	15 (60)	0.258
PT_05	19 (76)	23 (92)	0.247
Gross Score (0 to 5)		(p)[2]	
PT_T	3.32±0.99	4.44±0.58	<0.001

Data presented as N (%) or mean ± standard deviation.

[1] Chi-square test with Yates' continuity correction.

[2] Mann-Whitney *U*.

Table 1 presents the mean sum score of nine items of gross overall global assessment, which was 22.68±10.53 vs. 29.48±9.40 (p = 0.017). The items that showed the greatest differences were AVG_05 (knowledge of the procedure), with 2.36±1.58 vs. 3.40±1.41 (p = 0.013), AVG_08 (overall performance), with 2.12±1.27 vs. 3.04±1.43 (p = 0.026), and AVG_09 (final product quality), with 2.12±1.33 vs. 3.08±1.32 (p = 0.016).

The mean post-test sum score of five items was 3.32±0.99 vs. 4.44±0.58 (p<0.001), as shown in Table 1. The single item with the highest difference was about which region of the flap design should be incised first (PT_03), with a 44% vs. 92% correct answer rate (p = 0.001).

After baseline evaluation, the printed text group was exposed to the software and reevaluated for acquired knowledge and performance. Improvement in overall performance for each item was observed in the second evaluation, as shown in Table 2.

**Table 2:** Evaluation of the printed text group before and after CAL exposure.

Checklist			
Feature	Text (n = 25)	CAL (n = 25)	p-value[1]
Number of correct answers, n (%)			
Ckl_01	12 (48)	24 (96)	0.002
Ckl_02	11 (44)	24 (96)	<0.001
Ckl_03	12 (48)	24 (96)	0.002
Ckl_04	14 (56)	25 (100)	0.001
Ckl_05	10 (40)	23 (92)	<0.001
Ckl_06	11 (44)	23 (92)	0.002
Ckl_07	09 (36)	23 (92)	<0.001
Ckl_08	07 (28)	20 (80)	0.001
Ckl_09	08 (32)	20 (80)	0.002
Ckl_10	08 (32)	20 (80)	0.002
Gross Score (0 a 10)		(p)[2]	
Ckl_T	4.08±4	9.04±1.77	<0.001

Global Assessment			
Score (1 to 5)		(p)[2]	
Avg_01	3.12±0.83	3.88±0.83	0.001
Avg_02	2.48±1.16	3.76±0.83	<0.001
Avg_03	2.96±1.14	3.8±0.58	0.001
Avg_04	2.56±1.36	4.2±0.91	<0.001
Avg_05	2.36±1.58	4.2±0.87	<0.001
Avg_06	2.36±1.35	4.04±0.79	<0.001
Avg_07	2.6±1.48	4.0±0.82	0.002
Avg_08	2.12±1.27	3.92±1.08	<0.001
Avg_09	2.12±1.33	4.12±1.17	<0.001
Score (9 to 45)			
Avg_T	22.68±10.53	35.92±6.51	0.0001

Post-Test			
Features	Text (n = 25)	CAL (n = 25)	p-value[1]
Number of correct answers, n (%)			
PT_01	21 (84)	23 (92)	0.69
PT_02	22 (88)	25 (100)	0.25
PT_03	11 (44)	24 (96)	<0.001
PT_04	10 (40)	25 (100)	<0.001
PT_05	19 (76)	21 (84)	0.73
Gross Score (0 to 5)		(p)[2]	
PT_T	3.32±0.99	4.72±0.46	<0.001

Data presented as N (%) or mean ± standard deviation.

[1] McNemar.

[2] Wilcoxon.

All 50 students (100%) elected the software as the best method of teaching and would recommend its use to a friend if requested. They also reported a willingness to pay for the application if it were made available for download, even if the cost was twice that of the printed version.

When asked about the ability to safely perform a rhomboid flap without help from a teacher, 10 students (20%) said they would need only the printed text, 32 (64%) would need software and eight (16%) felt unable to perform the procedure alone, regardless of the supporting material.

## Discussion

CAL training is not intended to replace the actual experience or minimize the importance of teachers in regular classes with individualized feedback. Such assumption would be misleading, since we believe that the teacher's presence is essential for learning. CAL models are more efficient when provided individually to each student [19] as an ancillary tool to supplement learning. Both low-fidelity and high-fidelity training models - artificial or virtual reality models and animals or cadavers respectively - succeed in transferring knowledge and skills to surgical students [4]. Some authors suggest the superiority of high-fidelity models in specific training [20]–[22], which was not found in other studies [2], [23], where low-fidelity models were as effective as high-fidelity models. We chose to use a low-fidelity model to validate our method because of its efficiency and cost-effectiveness [24], [25].

Using the CAL concept and validating performance on an artificial model, we developed an efficient, easily deployable, and rapidly assimilated teaching and learning tool.

We found that students who used CAL showed better results than the printed text group, as reported in previous studies [26]. However, as with any learning method, we believe that training should be repeated continuously if information is to be retained [27], [28] because there is poor retention of content after 30 days even when a teacher is present during the learning process [29]. One potential advantage of CAL is that it also makes it easier to repeat the training while keeping the attractive advantage in cost-effectiveness as compared to high-fidelity models.

We also noted that certain items had greater between-group differences. Some items stood out on checklist evaluation, such as the flap orientation (p = 0.001), positioning (p = 0.023), setting (p = 0.005), and which of the sutures should be placed first (p = 0.002). This led us to believe that the multimedia animation method, despite good results for planning items as well, performed best in helping students understand actions that require motion. However, since no previous study was designed to assess these specific items, these results may be explained by our use of a study design that enabled the characterization of such differences.

On overall global assessment, the items with the greatest difference in favor of the CAL group were related to superiority of the final product (p = 0.016), overall performance (p = 0.026), and knowledge of major movements (p = 0.013), with no statistical differences for items related to tissue handling (p = 0.115), correct use of surgical instruments (p = 0.133) and dexterity (p = 0.084). This suggests that students who used the multimedia method showed much better performance and a much superior final flap product as compared to those of the printed-text group due to acquired knowledge, and not to greater skills or surgical dexterity.

Although not surprising, the fact that students performed differently was remarkable, because both teaching methods provided the exact same content. This ultimately suggests that methods that combine animation, audio and text may be much more effective than plain or even illustrated text when used in appropriate contexts.

The personal computing landscape is currently characterized by increasingly widespread access to content on mobile platforms such as smartphones and tablets. The availability and portability of knowledge can enhance learning, which seems particularly valuable as it can optimize the teacher's role in solving the specific difficulties of each student [30].

The software was built using Adobe Macromedia Flash software, which allows its use on different operating systems. With only minor modifications, it can be made compatible with mobile platform systems that have been experiencing exponential growth in recent years, led by the Android (Google, Mountain View, CA, USA) and iOS (Apple, Cupertino, CA, USA) systems.

## Conclusion

We successfully developed and validated a multimedia software application for teaching the rhomboid skin flap. Students who used CAL performed significantly better on objective parameters and subjective evaluation when compared to students exposed to a traditional printed textbook. Furthermore, participating students chose CAL as the most satisfactory method, which reinforces the applicability and acceptability of this training tool.
